# Determination of the Diagnostic Values of Asymmetric Dimethylarginine as an Indicator for Evaluation of the Endothelial Dysfunction in Patients with Rheumatoid Arthritis

**DOI:** 10.1155/2013/818037

**Published:** 2013-05-15

**Authors:** Dejan Spasovski, Arif Latifi, Bashkim Osmani, Svetlana Krstevska-Balkanov, Irena Kafedizska, Maja Slaninka-Micevska, Beti Dejanova, Sonja Alabakovska, Trajan Balkanov

**Affiliations:** ^1^Department of Rheumatology, University Clinical Centre, Skopje, Macedonia; ^2^Department of Hematology, University Clinical Centre, Skopje, Macedonia; ^3^Department of Preclinic Pharmacology, University Clinical Centre, Skopje, Macedonia; ^4^Department of Physiology, University Clinical Centre, Skopje, Macedonia; ^5^Institute of Preclinic Biochemistry, University Clinical Centre, Skopje, Macedonia

## Abstract

*Introduction*. To compare the diagnostic values of laboratory variables, to present evaluations of the diagnostic test for asymmetric dimethyl arginine (ADMA), rheumatoid factor (RF), C-reactive protein (CRP), and DAS_28_ index, and to define the effect of untreated rheumatoid arthritis on endothelial function. In order to determine whether ADMA changes depending on the disease evolution, ADMA was used as an indicator for endothelial dysfunction. *Methods*. Using an ELISA technology of DLD-Diagnostika-GMBH for the detection of ADMA, the samples of serum and urine have been examined in 70 participants (35 RA who were not treated, 35 healthy controls). RF was defined with the test for agglutination (Latex RF test) in the same participants. *Results*. Out of 35 examined patients with RA, RF appeared in 17 patients (sensitivity of the test, 51.42%). In 20 of the 35 examined patients with RA, we found the presence of ADMA (sensitivity of the test, 57.14%). Anti-CCP antibody was present in 24 examined patients with RA (sensitivity of the test, 68.57%). *Conclusion*. ADMA has equal or very similar sensitivity and specificity to RF in untreated RA (sensitivity of 57.14% versus 48.57%, specificity of 88.57% versus 91.42%) in the detection of asymptomatic endothelial dysfunction in untreated RA.

## 1. Introduction 

Rheumatoid arthritis (RA) is a disease which encompasses up to 1% of the whole population and is associated with an increased risk for onset and development of cardiovascular diseases (CVD). Data show that those patients have 30–60% increased risk for CVD in comparison with patients with osteoarthritis [1]. Patients with RA with disease duration of more than 10 years have increased risk of myocardial infarction [2] and more expressed process of atherogenesis because of the chronic systemic inflammation.

Circulatory markers of the systemic inflammation significantly increase the risk of cardiovascular morbidity and mortality in this group of patients, far more than traditional risk factors such as smoking, diabetes, and hypertension [3–9]. There are numerous analogues between RA and atheroclerosis, including macrophages, T-cell activation, unbalance between T helper 1 and T helper 2 lymphocytse, increased level of circulatory reactants of the acute phase and adhesion molecules, increased production of endothelins, oxidative radicals and increased neoangiogenesis in early but as well in long-lasting RA. Endothelial dysfunction occurs in the absence of manifest CVD and is not connected with traditional atherosclerotic risk factors [10–12].

### 1.1. Biomarkers for Assessment of Endothelial Dysfunction

Few classes of measurable serum proteins are used in the assessment of cardiovascular system:

1. biomarkers of myocyte injury: troponin I, troponin T, creatine kinase isoenzymes (CK-MB), and myoglobin;
2. biomarkers of myocyte stress: natriuretic peptides—A type (ANP), B type (BNP), adrenomedullin, midregional proadrenomedullin, and ST2;
3. biomarkers of remodeling: (MMPs) 1,2,7,8,9; (TIMPs) 1,2; procollagen type III;
4. Biomarkers of endothelial dysfunction: P-Selectin, scCAM-1, semi-carbazide sensitive amine oxidase (SSAO), vascular adhesion protein 1 (VAP-1), ADMA and SDMA, von Willebrandt factor, L-arginine, and nitric oxide metabolites (NO_*x*_);
5. biomarkers of inflammation: CRP, IL-1, IL-6, IL-10, TNF-*α*;
6. neurohormonal biomarkers: endothelin 1 (ET-1), big-endothelin 1 (Big ET-1).

Of the whole markers of the endothelial dysfunction, dimethyl derivatives of the amino acid L-Arginine incite greatest attention. There are two stereoisomer shapes of L-arginine, symmetric and asymmetric derivatives.

### 1.2. Symmetric Dimethyl Arginine (SDMA)

This is a methylated derivative of amino acid Arginine. It is eliminated from the body exclusively with renal excretion. Therefore, SDMA plasma concentration is tightly connected with the renal function. Determination of the plasma level of SDMA is important for assessment of renal failure [13–15]. 

### 1.3. Asymmetric Dimethyl Arginine (ADMA)

Synonyms (2S-2-amino 5-[(aminodimethylaminomethylen) amino]) pentanoic acid; N, N-Dimethylarginine; at C_8_H_18_N_4_O_2_ and natural chemical matter are normally present in plasma. They are metabolic products of continual processes of protein modification in cytoplasm in all human cells, tightly connected with essential amino acid L-arginine. ADMA interferes with L-arginine in the production of nitric monoxide (NO) which has key role in the normal endothelial function. NO is synthesised in endothelial cells with the enzyme endothelial nitric oxide synthetase (NOS) (EC 1.14.13.39). It has 3 isoenzyme forms: endothelial (eNOS), neural (nNOS), and in macrophages and in other immune cells (iNOS) involved in immune response. NO is activated through haemoglobin. 

Physiologic substrate, that is, precursor for NOS in this enzymatic process is L-arginine which is converted into NO and L-citrulline. NOS is inhibited by the endogenous arginine metabolite ADMA. Plasma level of ADMA is increased in RA.

ADMA is synthesised with protein methylation, that is, with posttranslational modification of arginine residues, in different proteins, mostly in cell nucleus. Methylation of the arginine residues is catalyzed by the group of enzymes called protein arginine N-methyl transferases (PRMTs). Basically, when proteins are proteolysed, free-methyl arginines are liberated.

The reaction is catalysed by the enzymes S-adenosyl-methionine (SAM) as well as (protein methylases type I and II) (PRMTs). They transfer one or more methyl groups from donor S-adenosyl-methionine to L-arginine with proteins or polypeptides. Both subtypes PRMTs have ability to methylate monomethyl arginine (MMA), so that type 1 asymmetrically dimethylates arginine and creates ADMA, while type 2 catalyses symmetric dimethylation of arginine and creates SDMA. ADMA and MMA have the ability to inhibit NOS but not SDMA. After synthesis, ADMA migrates to extracellular space and plasma. Human being generates about 300 *μ*mol/daily (about 60 mg/daily) of ADMA. Of this quantity, about 50 *μ*mol/daily is excreted in the urine. The elimination of ADMA is through urinary excretion after previous metabolization of the enzyme dimethylarginine dimethylaminohydrolase, dymethylargininasa, DDAH. There are 2 isoforms of DDAH_1_ and DDAH_2_ and which differ in the cell distribution. DDAH_1_ predominates in tissues that contain neural NOS, while DDAH_2_ is predominant in tissues with expression of endothelial NOS [16]. The main aim of this enzyme is degradation of methyl arginines, especially ADMA and MMA. The degradation of 80% of the daily production of ADMA is catalysed by DDAH [17]. 

## 2. Defining the Problem

RA is characterised with inflammation, key component in the onset and development of atherosclerosis. The inflammation, through activation of endothelial cells and through increase of the expression of leukocyte adhesion molecules, promotes proatherosclerotic environment. The endothelial dysfunction is an early preclinical marker for the onset of atherosclerosis and is often present in RA.

The endothelial dysfunction is closely connected with the inflammation, and therapeutic reduction of the inflammation leads towards its improvement. The assessment of the endothelial function is a useful tool in the identification and monitoring of the cardiovascular risk in patients with RA. Due to the fact that increased cardiovascular mortality is associated with RA, except the control of the activity of the disease, it is necessary to take measures, activities, and actions in the early detection and prevention of the cardiovascular risk. 

The systemic inflammation presented in RA is characterised with the activation of the vascular endothelium, leukocytes, and platelets. Incapability, that is, vasodilatation dysfunction of the arterioles known as endothelial dysfunction, is the first phase for the appearance of the atherosclerotic damage. The endothelial dysfunction is localised on the level of arterioles, <100 *μ*m in diameter, where the effect of NO is manifested, but it is forecondition for the appearance of atherosclerosis as ubiquitous disease of the middle-sized muscle arteries such as coronary, carotide, basilar, vertebral, and also with big calibre: aorta, iliacal, and arteries of the lower extremities.

The endothelial dysfunction is connected with several possible processes.

1. Changes in the formation of the extracellular matrix and synthesis of the components of the basal membrane.
2. Changes in the cell synthesis and liberation of vasoactive matters.
3. Changes in the processes of coagulation, platelet aggregation, and athession.
4. Proliferative processes of the smooth muscle cells and fibroblasts.
5. Dysbalance between endothelial relaxing and contracting substances.

### 2.1. Suggested Mechanisms for the Increased Plasma Level of ADMA in RA

(1) Decreased activity of DDAH, a key enzyme which regulates ADMA degradation but not SDMA, is able to increase the ADMA plasma level. ADMA is liberated during degradation of proteins that contain methylated arginine residues [18]. NOS is inhibited by ADMA.

(2) Activity of DDAH is reversibly proportional with the presence of tumour necrotic factor alpha (TNF*α*) [19–22], NOS, and S-nytrosylation. Because in RA quantities of TNF*α* oxygene radicals and 3-nitrotirosine are increased, it is hypothesised that the selective increase of ADMA plasma level in RA is the result of decreased DDAH activity in plasma. The chronical presence of proinflammatory cytokines as IL-1a, IL-1b, IL-6 and TNF*α*, and CRP from the synovial tissue directly influences the systemic endothelial dysfunction. Treatman with anti-TNF*α* in the 12th week significantly improves the endothelial vasodilatation of the brachial artery [23].

(3) Second possibility is that oxidative stress connected with RA can increase formation of ADMA through increased expression of protein arginine 1 N-methyltransferase [24]. Substrates of these argine 1 N methyltransferases are proteins with side-chain arginine guanidine groups.

(4) Third possibility is that the inflammatory synovia in RA is with increased proliferation and potent apoptosis of the vascular endothelial cells [25]. The cultivated endothelial cells liberate more ADMA than SDMA [26]. Increased endothelial cell turnover in RA with successive liberation of ADMA during catabolism of the proteins results in increased level of ADMA in RA.

(5) Hypoxia in the inflamed synovia in RA decreases expression of DDAH [27]. Decreased DDAH activity increases the concentration of ADMA. 

(6) The tendency towards insulin resistance is noticed in patients with RA, but positive association between plasma level of ADMA and insulin resistance is noticed in healthy individuals with different levels of insuline sensitivity [28]. This phenomenon is hypothetically connected with increased liberation of ADMA.

(7) The role of homocysteine as a risk factor for CVD is through the inhibitory effect of the regulatory production of DDAH in the body. It is quickly regulated with vitamin B supplements. High endothelial LDL cholesterol increases the level of ADMA which in return inhibits the production of NO necessary for vasodilatation.

Increased level of ADMA is found in patients with homocysteinemia [29], coronary disease [30], peripheral arterial occlussive diseases [31], pulmonary hypertension [32], and as a result of smoking [33] and diabetes [34]. Common thing for all these conditions is that ADMA is endogenous inhibitor of NOS. New perspective and cross-sectional studies indicate that the increased level of ADMA is a risk factor for cardiovascular conditions and lethal outcome [35]. ADMA is significant as a new cardiovascular risk factor in RA.

ADMA activity is low in serum and is increased in the presence of endothelial dysfunction due to the presence of inflammation in RA. It is a very sensitive indicator of endothelial damage in comparison to the invasive and noninvasive functional measurements. The use of ADMA is a relatively simple, cheap, fast, and nonivasive method in detection of the early stadium of diesease and followup of the endothelial disorder. Therefore, its presence would be quantified with the level of inflammation in RA which correlates with the disease activity.

## 3. Aims of the Study

1. To determine initial presence of ADMA in patients with RA.
2. To examine the connection between plasma ADMA level and the activity of the disease with its characteristics. To investigate whether circulatory factors of NO metabolism, ADMA, reflect on clinical characteristics of RA.
3. To determine whether mediators of the inflammatory reactions in RA influence microvascular, endothelial NO metabolism.
4. To confirm the statement that the appearance of the subclinical atherosclerosis in RA is connected with the accumulation of the endogenous inhibitor of nitric monoxide synthetase and plasma level of ADMA.
5. To determine correlations among plasma level of ADMA with the duration of disease, index of the strength of the disease (DAS-28), age of the patient and duration of disease, and evolution of disease before the beginning of treatment.
6. To determine correlations between plasma level of ADMA and reactants of the acute phase.
7. To determine whether seropositivity in RA influence plasma level of ADMA.
8. To determine whether there is correlation between ADMA and anticyclic citrullinated peptide antibodies (anti-CCP_2_) from the second generation.
9. On the base of the values of examined parameters that should be followedup and their correlation to determine whether biomarker would be the most adequate not only for early detection of endothelial damage but also for the best clinical, prognostic, and economic end-effect.

## 4. Material and Methods

In the patients examined for this study, the diagnosis of the disease was established on the basis of revised diagnostic criteria for the classification of rheumatoid arthritis, suggested in 1987 by the American Association for Rheumatism (ARA) [36]. In order for a patient to be diagnosed with rheumatoid arthritis, he or she must fulfill at least four out of seven criteria. Criteria from one to four are present for at least six weeks. The study involved 35 patients (female 27, male 8) suffering from RA and 35 healthy subjects (female 17, male 18) from the control group. Their average age was 55.57 years (±5.68) (39–65 years) in the group with RA and 45.1 years (±11.37) (29–65 years) in the control group. The average duration of the disease in months from the beginning was 42.86 (±44.12) in the interval of 5–157 months. None of the patients had been previously treated with oral corticosteroids. The rest of the patients refused to use other medicines before taking the examinations; Table 1.

### 4.1. Including Criteria

In this study, patients with RA, age 18–65, newly diagnosed and not treated would be included.

### 4.2. Excluding Criteria

From the study all patients/individuals with diseases or conditions that could directly or indirectly influence changes of results (diagnosed in the course of the study would be excluded), like the following:

1. patients with past history for disease of the spleen, liver damage, renal, hematological, cardiological, neurological, and lung injuries, autoimmune diseases, AIDA, and age <18 years;
2. patients suffering from conditions that affect lipid profile, such as smoking, diabetes, hypothyreosis, Cushing's syndrome, and obesity (body mass index >30, with family history of dyslipidemia);
3. patients with acute infections, malignant diseases, febrile conditions;
4. patients previously treated with some basic drug for RA, including CSs and NSAIDs;
5. patients with hypertension, uric gastritis, urinary infections, systemic lupus erythematosus, mixed connective tissue disease, vasculitis, systemic scleroise, primary and secondary Raunaud phenomena, periferal vascular diseases, and congenital CVD;
6. patients treated with antihypertensive, antidiabetic, and cardiologic therapy, drugs for decrease of the lipid profile, oral contraceptives, estrogens, progesteron, and tocopherol (vitamin E);
7. patients previously blood transfused;
8. patients in whom in 0-time would be found increased level of glycose, degradational products, like serum and urine creatinin, serum urea, hypertension, and changes in hematological parameters or enzymatic status.

All patients took part in this study voluntarily, so the ethical criterion was not breached during our work.

### 4.3. Clinical Evaluation of Disease Activity

Changes in the evolution of disease, that is, clinical improvements, would be assessed through suitable indexes of activity (clinical parameters) (Disease Activity Score-DAS_28_) [37–40]. The index uses mathematical formula in order to get unique composite quantitative score composed of palpatory painfully sensitive joints (maximal score 28), swollen joints (maximal score 28), Westergren ESR, and patients global evaluation for disease activity (0–100 mm Visual Analogue Scale-VAS). 

DAS_28_ index ranges from 0 to 10, and score below 3.2 qualifies the disease as low active.

DAS_28_: 0.56 0.56 √0–28 palpatory painfully sensitive joints + 0.28 √0–28, swallen joints +0.70 (log⁡_*n*_ ESR) + 0.014 (global estimation of the patient for disease activity: VAS 0–100).

### 4.4. Biochemical Laboratory Examination

For a clinical assessment of the basic disease, the following laboratory variables needed to be measured: haemogram and differential haemoalysis, reactors of acute phase, anti-CCP_2_ antibody, C-reactive protein (CRP), rheumatic factor (RF), alkaline phosphatase (AP), aspartate aminotransferase (AST), alanine aminotransferase (ALT), creatinine kinase (CK), lactate dehydrogenase (LDH), urea and creatinine in serum, and Asymmetric dimetil L-arginine (ADMA).

### 4.5. Determination of the Activity of Asymetric Dimethyl L-Arginin (ADMA): ELISA Method (DLD Diagnostics, GMBH) Enzyme Immunoassay

For quantitative determination of the endogenous Asymetric dimethyl L-arginine (ADMA) in serum or plasma. 

Principle: ADMA is adhered in solid phase on microtiter plate. ADMA standard, samples, and positive control are preacylated, in combination with rabbit anti-N-acyl-ADMA, incubated for 15–20 h at 2–8°C. After that they are washed, and second antibody is added, anti-rabbit IgG, conjugated with peroxidase. After 1^−^hour incubation a room temperature they are washed. In order to achieve colour, tetramethylbenzidine is added as a substrate solution (TMV). After 20–30 minutes incubation, it is stopped with adding sulphur acid. The absorbtion is read on 450 nm on automatical microtitar reader. With the standard figureis determined ADMA concentration. Basically, the quantity of antibodies adhered in the solid phase of ADMA is reversely proportional to the concentration of ADMA in the samples.

Refererent values: ADMA in serum 0.4–0.75 micromol/L.

Determination of the C-reactive protein (CRP) and rheumatoid factor (RF) with test for agglutination (Latex CRP test). (BioSystems S. A. Reagens & Instruments Costa Brava 30, Barcelona, Spain).

Refererent values are +>6 mg/L CRP in serum; +>8 mg/L for RF in serum.

Determination of anticyclic cetrullinated peptide antibodies (anti-CCP_2_) with semiquantitative/qualitative ELISA technique, based on the detection of IgG autoantibodies in human serum or plasma, directed towards synthetic cyclic citrulated peptides (CCPs) that contain modified arginine residues. The DIA-STAT anti-CCP (Axis-Shield Diagnostics). 

Refererent values are +>1.26 positive values for anti-CCP in serum.

Determination of the erythrocyte sedimentation rate (ESR) with quantitative method of Westergren with normal values for males is 7-8 mm, for woman 11–16 mm.

## 5. Statistical Analysis 

Predictive values for positive and negative results and accuracy of examined marks were defined with the test of sensitivity and specificity. Analysis of the structure of numeric series is made by the measures of the central tendency (average) and measures of dispersion (standard deviation); analysis of the structure of the attributive statistical series is made by coefficients of correlations and proportions; analysis of correlation between numeric statistical series is made by Pearson's coefficient of correlation-*p*; analysis of correlation between attributive statistical series is made by Pearson's *χ* ^2^-test. To test the significance of differences between two arithmetical medians that is, proportions Student's *t*-test is used and a Wilcoxon-matched test for independentexamples. To test the significance of differences among three or more arithmetical medians, analysis of variance (ANOVA) is used. A *P* value between 0.05 and 0.1 was taken as statistically significant. The results would be statistically processed with the following statistical methods and shown with statistical package Statististica 7.0.

## 6. Results

Of all the examined patients with RA, ADMA is detected in 20 patients (57,14%), about acute phase reactant, RF was detected in 17 patients (48.57%), anti-CCP antibody in 23 patients (65.71%), CRP in 14 patients (40%), and sedimentation in 27 patients (77.14%). In the healthy control group, RF was present in 2 patients (5.71%). DAS_28_ is present in 28 patients with RA (80%). Among 18 RF negative patients, 7 patients (38.88%) were ADMA positive. Among 17 RF-positive RA patients, presence of ADMA was found in 13 (76.47%) patients. In the healthy control group, 4 patients (11.42%) were ADMA positive; Table 2.

Diagnostic value of ADMA in patients with rheumatoid arthritis (RA) sensitivity, specificity, and predictive values of the positive and negative tests as well as their precision is shown in Table 3.

ADMA has equal.or very similar sensitivity and specificity from RF in untreated RA (sensitivity 57.14% versus 48.57%, specificity 88.57% versus 94.28%) in the detection of RA. 

### 6.1. Association between Asymmetric Dimethylarginine and Disease Activity Score (DAS_28_) in Patients with Rheumatoid Arthritis

Among 35 patients with RA, DAS_28_ was replaced in 29 patients (82.85%). In 17 seropositive RF patients, replacement of DAS_28_ was detected in 16 patients (94.11%). Of these 16 DAS_28_ patients, 9 were ADMA positive (56.25%). Among 18 seronegative RF patients, replacement of DAS_28_ was found in 14 patients (77.77%). Of these 14 DAS_28_ patients, 5 were ADMA positive (35.71%), and their M±SD was 6.03 ± 0.21. 

Seropositive RF patients have bigger ADMA value of circumference than seronegative RF patients (13,16 (±0,40) versus 11,22 (±0,72) and bigger DAS_28_ index of intensity of disease (6.03 ± 0.21) versus (3.75 ± 0.85). Between these two groups of ADMA), there is no statistical connection (*P* = 0.74); see Table 2. 

There was no statistical connection using the Wilcoxon-matched test between the DAS_28_ index in RF seropositive and seronegative patients (*P* = 0.48) and between two groups of DAS_28_, ADMA positive patients and seronegative patients (*P* = 0.33).

**Figure 1:**
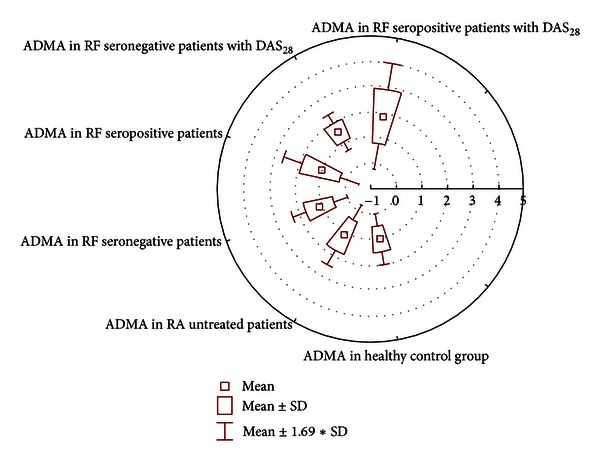
Distribution of asymmetric dimethylarginine (ADMA) in serum (0.4–0.75 micromol/L).

1. Statistical relation was found using the Wilcoxon-matched test between ADMA in RA and age, duration of disease, RF, CRP, sedimentation, and morning rigid, in the same group for *P* < 0.05 (ADMA vs age *P* = 0.000; ADMA vs duration of disease, *P* = 0.000; ADMA vs RF, *P* = 0,015; ADMA vs CRP *P* = 0,030; ADMAvsSedimentation, *P* = 0,000).
2. There was no statistical connection using the Wilcoxon-matched test between ADMA in RA and healthy control group for *P* < 0.05 (*P* = 0.064), Figure 1.

## 7. Discussion

Not only the ever growing prevalence of the CVD in RA, but also the use of drugs that change the disease activity in disease treatment contributes to the deepening of the investigations towards finding easy, cheap, and practical screening method, in order to detect the early stadium of disease of the endothelium.

There are several biomarkers as indicators for endothelial dysfunction such as endothelial vasoactive substances, leukocyte athesion substances, proinflammatory cytokines, prostaglandins, reactants of the acute phase, hormones, enzymes. Everyone has its own importance in the functioning of the blood vessels. ADMA is the unique substance on which the multifunctional role of NO depends, but as endogenous antiatherogenic molecule. Direct effect on the autoregulation of the normal myogenic activity and vasomotion (potent vasodilator effect), athession (inhibition of the platelet aggregation, inhibition of athession of the monocytes and leukocytes in normal vascular endothelium), proliferation (inhibition of proliferation of vascular smooth muscle cells), with basic aim achievement and maintenance of trans musculare, capillary pressure. ADMA has indirect effect on the process of atherosclerosis: it decreases vascular liberation of superoxide radicals involved in the inflammatory and cytokine processes and inhibition of LDL oxydation.

Until recently ADMA was detected with liquid chromatography, making it complex, inaccessible, technologically dependent, and expensive. With the introduction of ELISA method in the detection of ADMA, determination becomes more accessible and more practical.

ADMA plasma level is increased in RA without CVD or presence of risk factors. There is strong association between ADMA and anticyclic citrulated peptide antibodies (anti-CCP_2_) from the second generation in early RA without cardiovascular risk factors as a result of the activity of disease and inflammatory activation. Our case shows that there is association between ADMA and anticitrulated peptide antibodies (anti-CCP_2_) in early RA [41–44]. ADMA plasma level shows dependence on duration and disease evolution. Seropositivity in RA has influence on ADMA plasma level. It shows in our example that patients with presence of RF with DAS_28_ index of disease have higher ADMA induction than seronegative patients with DAS_28_. Statistical connection between disease duration and presence of ADMA show that untreated RA works an endothelium as one of visceral appearance of the disease. 

 Endothelial dysfunction has been suggested as an early event in the atherogenic process and as a novel predictor of CHD events; more recent longitudinal studies in the CHD arena provide some support for this proposition [45]. Several studies employing various “direct” measures of vascular function such as pulse wave analysis [46], flow-mediated vasodilation [47], and venous occlusion plethysmography [48] confirm endothelial dysfunction in RA patients. Where examined, such dysfunction has been linked to systemic inflammatory markers. Vaudo and associates [49] have provided more recent evidence for endothelial dysfunction in young to middle-aged patients with low disease activity (disease activity score), DAS ≤3.2, and noted a strong association to average CRP levels. This finding agrees with the earlier observation of an early excess risk of vascular events in RA patients. Elevated LDL cholesterol also was an independent correlate to impaired vascular function in RA patients in the latter study. Other more recent studies also show impaired vessel stiffness with the technique of pulse wave velocity [50], and we have shown impaired forearm microvascular function using laser Doppler imaging in patients with RA in parallel with a greater systemic inflammatory response [51].

More recent longitudinal studies report improved endothelial function after antitumor necrosis factor (TNF)-*α* therapy [52, 53], but this benefit seems to be transient and linked to the pattern of change in systemic inflammatory markers on TNF blockade. Treatment with disease-modifying antirheumatic drugs (DMARDs) also improves endothelial function in patients with RA [48], so the mechanism employed to achieve inflammatory control may not be crucial to the improvement in endothelial function.

## 8. Conclusion

 ADMA has high sensitivity in the detection of asymptomatic endothelial dysfunction in untreated RA. The practical significance of the determination of ADMA plasma level in everyday clinical practice results form the fact that the doctor, according to the results of its concentration could change not only diagnostic but also therapeutic tactics. With the followup of the dynamics of changes of concentration of ADMA, and they could judge for the nature and development of the disease and its outcome. These possibilities make determination of ADMA plasma level practically important for on-time information to the doctor, considering disease nature and its severity.

## Figures and Tables

**Table 1:** Patients characteristics examined in the study.

	RA no. 35 Value (M ± SD)	Control healthy group no. 35 Value (M ± SD)
Male/female relation	8/27	18/17
Middle average age (years)	55.57 (±5.68) (39–65)	45.10 (±11.37) (29–65)
Middle duration (time) of disease (month)	42.86 (±44.12) (5–157)	0.00 (±0.00) (0.00-0.00)
Previous therapy with oral corticosteroids (no. of patients)	0	0
Previus therapy with methotrexate (no. of patients)	0	0

**Table 2:** Distribution of ADMA, reactant of acute phase and other laboratory variables in patients with RA and healthy control group.

	Ra not treated group no. 35 Value (M ± SD)	RA^sero−^ no. 18 Value (M ± SD)	RA^sero+^ no. 17 Value (M ± SD)	Control healthy group no. 35 Value (M ± SD)
	Positive/negative	Positive/negative	Positive/negative	Positive/negative
ADMA +>0.75 *μ*mol/L	20/15 08.10 (±0.75) (0–210)	7/11 11.22 (±0.72) (0–110)	13/4 13.16 (±0.40) (0–210)	4/31 11.75 (±0.22) (55.20–105.33)

Sedimentation +≥16	25/10 46.43 (±41.79) (1.0–110)	11/7 40.70 (±37.63) (1.0–110)	13/4 51.38 (±37.37) (1.0–110)	3/32 7.31 (±7.10) (1.0–31)

Anti CCP_2_ ≥1.26	24/11 1.82 (±0.71) (0.91–2.0)	12/6 1.45 (±0.48) (0.91–1.5)	11/6 1.76 (±0.80) (0.91–1.9)	1/34 0.84 (±0.09) (0.90–1.27)

RF +≥8 IU/mL	18/17 335.09 (±515.11) (0.00–512)	0/18 0.00 (±0.00) (0.00-0.00)	17/0 601.67 (±622.68) (32–1024)	3/32 11.69 (±40.69) (0.00–128)

DAS_28_ +≥3.2	29/6 5.81 (±1.44) (1.76–6.01)	14/4 5.30 (±1.55) (1.72–6.01)	16/1 4.01 (±1.29) (1.39–5.81)	0/35 0.00 (±0.00) (0.00-0.00)

**Table 3:** Diagnostic values of ADMA, and other labaratory variables in patients with RA.

	Sensitivity %	Specificity %	Predictive values for the positive test %	Predictive values for the negative test %	Accuracy %
ADMA RA no. 35	57.14	88.57	83.33	32.60	72.85
ADMA RA^−^ no. 18	38.88	88.57	63.63	26.19	71.69
ADMA RA^+^ no. 17	76.47	88.57	76.47	11.42	84.61
Sedimentation RA no. 35	71.42	91.42	89.28	23.80	81.42
Sedimentation RA^−^ no. 18	61.11	91.42	78.57	17.94	81.13
Sedimentation RA^+^ no. 17	76.47	91.42	81.25	11.11	86.53
Anti CCP antibody RA no. 35	68.57	97.14	96	24.44	82.85
Anti CCP antibody RA^−^ no. 18	66.66	97.14	92.30	15	86.79
Anti CCP antibody RA^+^ no. 17	64.70	97.14	91.66	15	86.53
RF RA No35	51.42	91.42	85.70	34.69	71.42
RF RA^−^ no. 18	0	91.42	0	36	60.37
RF RA^+^ no. 17	100	91.42	85	0	94.23
DAS_28_ RA no. 35	82.85	100	100	14.63	91.42
DAS_28_ RA^−^ no. 18	77.77	100	100	10.25	92.45
DAS_28_ RA^+^ no. 17	94.11	100	100	2.77	98.07
